# Impact of Therapist Breaks on Patients with Borderline Personality Disorder: A Review

**DOI:** 10.1192/j.eurpsy.2025.2142

**Published:** 2025-08-26

**Authors:** I. Gözel, I. İnan

**Affiliations:** 1 Psychology, Ankara Bilim University; 2 Psychology, Hacettepe University, Ankara, Türkiye

## Abstract

**Introduction:**

Borderline Personality Disorder (BPD) is characterized by abandonment schemas, impairments in emotion regulation, and attachment issues. The literature shows significant evidence that psychotherapy is effective in the treatment of BPD in addition to pharmacotherapy. The therapeutic relationship established with the patient is a crucial component of psychotherapy processes and plays a vital role in the prognosis of the disorder. Working on the therapeutic relationship is essential to maintain an effective psychotherapy process in BPD treatment. The therapeutic alliance is at the core of the treatment’s success. When a therapist takes a break from the therapy process (due to reasons such as vacation, childbirth, or loss), ruptures may form in the therapeutic relationship with patients diagnosed with BPD.

**Objectives:**

This review compiles and synthesizes findings from empirical and case studies on the effects of breaks in psychotherapy treatment for BPD patients.

**Methods:**

PsycArticles, Web of Science, PubMed, and Google Scholar were reviewed.

**Results:**

Immediately following a therapy break, BPD patients often experience intense feelings of anger, anxiety, and frustration. At the same time, in the long term, this can lead to distrust in the therapist and weaken the therapeutic relationship. Moreover, acting-out behaviors tend to increase during this period. For inpatient patients, this can be observed as non-compliance with clinic rules or using verbally inappropriate expressions.This break in the therapy process activates the abandonment schema, which is a core characteristic of BPD. Patients with borderline personality organization return to the dysfunctional coping strategies they had previously developed.

**Conclusions:**

These results highlight several important areas of discussion for clinical practice and research. Firstly, BPD patients should be informed about upcoming therapy breaks and prepared for the absence, and expectations should be discussed openly. Secondly, after the break, working on the therapeutic relationship, mistrust, the activated abandonment schema, and negative emotions directed toward the therapist will contribute to the healing process. Thirdly, given the risk of suicide linked to impulsivity and acting out, the therapist should develop an action plan. Fourthly, maintaining the therapeutic framework is one of the most critical steps in the recovery of BPD patients, so it is crucial to preserve the framework even during therapy breaks. Lastly, due to the gap in the literature on this subject, there is a need for more empirical studies and research on how to repair potential ruptures in the therapist-patient relationship that occur during breaks.

**Disclosure of Interest:**

None Declared

